# The Petri dish under the ice: permafrost pathogens and their impact on global healthcare and antibiotic resistance

**DOI:** 10.1097/MS9.0000000000002650

**Published:** 2024-10-11

**Authors:** Muhammad M. Saleem, Naz Elahi, Roha Athar, Areeba Gul, Mariam Adil, Aayat Ellahi, Haider Kashif, Moussa Hojeij

**Affiliations:** a Department of Medicine, Dow University of Health Sciences; b Department of Medicine, Karachi Medical and Dental College; c Department of Medicine, Jinnah Sindh Medical University, Karachi, Pakistan; d Department of Medicine, Lebanese University, Beirut, Lebanon

**Keywords:** ancient bacteria, antibiotic resistance, climate change, epidemic, permafrost thawing

## Abstract

**Background:**

A shallow active layer of soil above the permafrost thaws during the summer months which promotes microbial growth and releases previously confined pathogens which result in bacterial epidemics in circumpolar regions. Furthermore, these permafrost sources harbor several antibiotic resistance genes (ARGs) which may disseminate and pose a challenge for pharmacologists worldwide.

**Aims:**

The authors examined the potential association between climate change-induced permafrost thawing, and the resulting release of antibiotic-resistant pathogens, as well as the potential impact this can have on global healthcare systems in the long run.

**Methodology:**

A cursory abstract screening was done to rule out any articles that did not have to do with viral pathogens caused by melting permafrost. Articles that were not available in English or that our institutions library did not have full-text access were weeded out by a secondary screen.

**Results:**

A comprehensive analysis of 13 relevant studies successfully revealed a wide variety of bacterial genera, including *Staphylococcus spp.*, *Pseudomonas spp.*, *Acinetobacter spp.*, and *Achromobacter spp.*, along with a total of 1043 antibiotic resistance genes (ARGs), with most pertaining to aminoglycosides and beta-lactams, offering resistance via diverse mechanisms such as efflux pumps and enzymatic modifications, within the permafrost isolates. Additionally, mobile genetic elements (MGEs) housing antibiotic resistance genes (ARGs) and virulence factor genes (VFGs), including plasmids and transposons, were also discovered.

**Conclusion:**

Permafrost thawing is an underrated healthcare challenge warranting the need for further articles to highlight it alongside concerted efforts for effective mitigation.

## Introduction

Highlights

- Climate change induces permafrost thawing, which releases trapped pathogens.
- These permafrost pathogens cause bacterial epidemics in circumpolar regions.
- Several ARGs, offering resistance via several mechanisms, have also been discovered in permafrost.
- The presence of ARGs in permafrost is evidence that antibiotic resistance is an ancient phenomenon.
- Mobile genetic elements such as plasmids and transposons were also discovered.

Permafrost occupies about 16% of the global terrestrial area, but contributes to ~50% of the soil organic carbon stock in terrestrial ecosystem, with the permafrost zone in the Northern Hemisphere storing as much as 1014 Pg (1 Pg=10^15^ g) carbon in the top 3 m of the soil^1^. When temperatures rise, eventually thawing permafrost, the enzymes concentrated in the active surface of soil, hydrolyze organic matter to promote microbial growth, and this accelerates the decomposition reactions they catalyze^1,2^. Furthermore, upon melting of frozen soil, cold-adapted microbes are presented with a higher soil water content from the liquefied ice, and improved nutrient content from growing vegetation^3^. Consequently, microbial growth and fermentation release CO_2_ and CH_4_ into the atmosphere, which may potentially induce various catastrophic effects on human health, including the onset of asthma, reduced lung function, and can stimulate a carbon-positive loop^2,4^.

It has thus become crucial for scientists to find ways to combat climate change-driven deterioration and the potential effects it may have on the healthcare system. Researchers might contribute to this cause by focusing on the mechanisms of action of permafrost-preserved pathogens. Intriguingly, it has been proposed that the process by which these pathogens are preserved in frozen ground includes the lowering of their freezing point due to high ionic strength within pore water. This property of pore water also preserves the cell viability of these microbes^2^. Moreover, cold environments slow down cell metabolism and the rates of nutrient uptake, thereby allowing cryophiles to conserve energy. Permafrost is thus an ideal reservoir for pathogenic organisms^5^.

Furthermore, the melting of permafrost that is ~30 years old poses a considerable risk to global public health, due to its potential to release pathogens that may have been previously confined there. Some of these microbial pathogens include herpesvirus, rotavirus, and enterovirus, which are known to sustain their viability after freezing and thawing. These various, diverse permafrost microbes and their release into the atmosphere, subsequent to increased global warming, have led to increasing outbreaks of bacterial and viral epidemics in circumpolar regions^6,7^.

Thermokarst lakes form as the results of ice-rich permafrost thawing, and act as important water resources in cold regions. The results of a recent study have revealed that both sediment and water of the thermokarst lakes in the Yellow River Source Area harbor diverse antibiotic resistance genes, most of which belong to efflux pump, suggesting that the microbial resistances are intrinsic and ubiquitous in this pristine system^8^. A detailed genome analysis of the *Acinetobacter lwoffii* bacteria isolated from permafrost thaw, has shown that the pathogen is resistant to widely used antibiotics such as streptomycin, spectinomycin, chloramphenicol, and tetracycline^9^. It has been hypothesized that these pathogens would not be detected by modern tests, due to the different genetic makeup of archaebacteria. The same reasoning can apply to a potential reason as to why they are resistant to most commonly used drugs. These findings pose a cause of concern, as modern pharmacology systems are not well adapted to treating these diseases, and global warming can only be slowed down, at best.

In addition to the direct consequences of the thawing of permafrost on global healthcare by means of microbes, there is the emergence of indirect effects as well. Permafrost thawing poses the problem of the inavailability of medical services around the Arctic region due to transportation issues, as well as the destruction of existing healthcare facilities due to ground degradation^10^. However, our review focuses on the direct effects of pathogens liberated by melting permafrost. Since there is scarce literature available on the aforementioned problem, our aim was to gather all accessible information to proficiently analyze the current situation. By evaluating existing research, determining the limitations and strengths of previous articles, and discerning what can be learned from further studies, we examined the potential association between the causes of permafrost thawing, including primarily a global warming, climate change-induced positive carbon feedback loop, and the resulting release of antibiotic and antimicrobial resistant pathogens, as well as the potential impact this can have on global healthcare systems in the long run.

### Methodology

**Figure 1:**
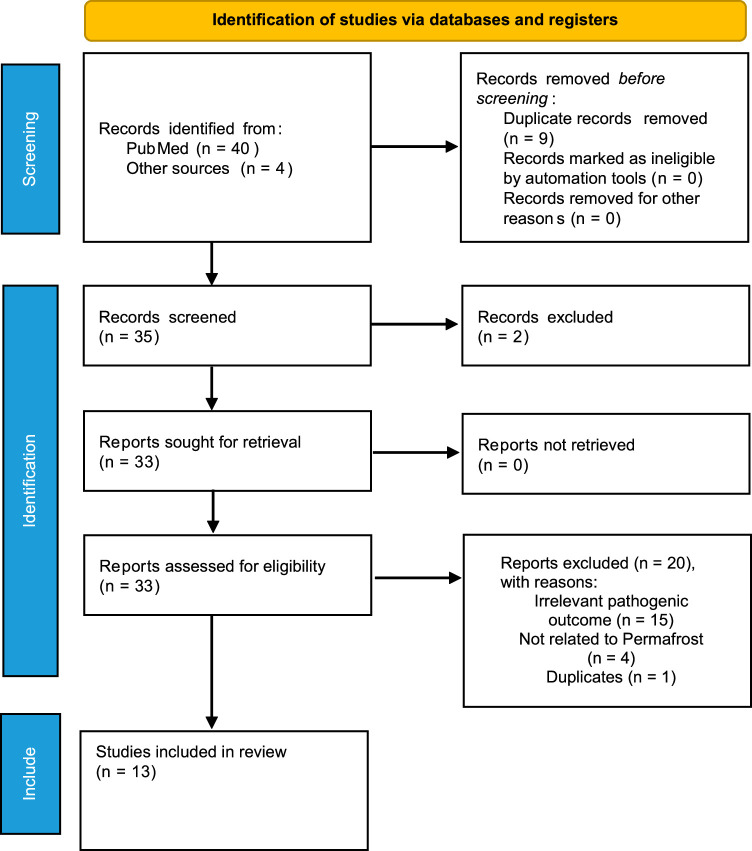
PRISMA study selection flow diagram.

The Preferred reporting Items for Systematic Reviews and Meta-Analyses (PRISMA) standards^11^ were followed for conducting this narrative review. The PRISMA flow diagram (Fig. 1) highlights the entire literature search and study selection process.

### Search strategy

Through 18th December 2023, an independent reviewer searched the PubMed, Cochrane, MEDLINE, and Embase central databases. There were no time or language constraints. The search approach combined MeSH terms with the Boolean operators ‘AND’ and ‘OR’ to discover the distinct keywords. A combination of the search terms used were ‘permafrost’ AND ‘pathogenicity’ AND ‘healthcare’.

### Inclusion criteria

The relevance of all research found through keyword searches in databases was investigated. Material that included the primary outcome of viral and bacterial pathogenic infections, and had full text available, was included based on an abstract screening. Due to the limited number of publications currently available, there were no restrictions on the kind of publication, quality, research technique, or risk of bias.

### Exclusion criteria

A cursory abstract screening was done to rule out any articles that did not have to do with bacterial and viral pathogens caused by melting permafrost. Articles that were not available in English or that our institutions library did not have full text access were weeded out by a secondary screen.

### Study selection

Two independent reviewers initially short-listed studies based on their title and abstract, after which they were imported into Endnote, where duplicates were discarded. The remaining articles were then evaluated at the full-text level using the aforementioned criteria. In case of any discrepancies, a third independent reviewer was consulted. All studies were finalized after a consensus was reached between all authors.

### Data extraction

Data extraction was performed independently by two authors who collected and categorized the data into the following main categories: author and year of publication, permafrost location, bacteria identified, antibiotic resistance genes (ARGs), plasmids, and transposons present. In case of missing data or lack of clarity, authors were emailed for assistance. Extracted data was reviewed, corrected, and finally organized into a comprehensive table.

## Results

### Study selection

A meticulous search of databases identified a total of 35 studies among which 2 were excluded after evaluation of their title and abstract. The remaining 33 studies were assessed for their inclusion eligibility via a full-text review, which excluded 20 studies (irrelevant pathogenic outcome *n*=15, unrelated to permafrost *n*=4, and duplicate =1). Finally, 13 articles satisfied the inclusion criteria and were pooled together in the systemic analysis^8,12–23^. The complete study selection procedure is demonstrated in a PRISMA Flowchart (Fig. 1).

### Study characteristics

Our narrative review presents the results of a comprehensive literature search conducted to investigate antibiotic resistance genes in permafrost isolates collected from diverse locations around the globe, including Siberia, interior Alaska, and other regions. The year of publication of short-listed studies spanned from 2009 to 2022. A comprehensive analysis of 13 relevant studies successfully revealed a wide variety of bacterial genera, including Staphylococcus spp., Pseudomonas spp., Acinetobacter spp., and Achromobacter spp., along with a total of 1043 ARGs, with most pertaining to aminoglycosides and beta-lactams, within the permafrost isolates. Significantly, the aforementioned genes demonstrated resistance to a diverse array of antibiotic phenotypes, as outlined in Table 1.

**Table 1:** Study characteristics.

Author - year	Permafrost SITE/location	Identified bacteria	Number of ARGs detected in permafrost	Name of ARGs detected in permafrost	Resistance Mechanisms (most common)	Phenotype in ARGs detected- antibiotic name	Mobile elements found in permafrost isolates	Plasmids	Transposon	Plasmid borne ARGs	Transposon borne ARGs
Kashuba 2017^13^	Mammoth Mountain in Siberia	Staphylococcus warneri species (designated MMP1) Staphylococcus hominis species (designated MMP2)	Six in MMP2	Aminoglycoside: aph(3’)-III, ant(6)-la, beta-lactams: blaZ, MLS: msr(A), mph(C) and phenicol: cat(pC194).	N/A	Aminoglycosides, beta-lactams, MLS, phenicol	Plasmids	Two contigs (#23 and #24) in MMP1 3 contigs (#51; #53 and #54) in MMP2	N/A	N/A	N/A
Petrova 2009^20^	Eastern-Siberian Sea	P. psychrophilus	N/A	N/A	N/A	N/A	Plasmid and transposon	pKLH80	Tn5080	orfX and the entire antibiotic resistance area of Tn5080(streptomycin and tetracyclin resistance genes)	Streptomycin (strA-strB) and tetracycline (tetR and tet(H)) RG
Petrova 2011^21^	Near Tiksi, Coast of Laptev Sea.	Pseudomonas sp.	N/A	N/A	N/A	N/A	Transposon	N/A	Tn5045	N/A	aadA2 (streptomycin/spectinomycin resistance gene)
Rakitin 2021	Kolyma lowland-Republic of Yakutia-Russia	Acinetobacter lwoffii	29	Cat, blaOXA-134, macAB-tolC, aadA27, tet(H), cflA	Chloramphenicol acetyltransferase OXA-134 family class D β-lactamase Drug efflux ABC-type transporter	Chloramphenicol acetyltransferase (cat) OXA-134 family class D β-lactamase ( blaOXA-134 ) Drug efflux ABC-type transporter ( macAB, tolC) Streptomycin- spectinomycin, 3”-adenylyltransferase ( aadA27) Tetracycline efflux MSF transporter [tet(H)] Drug efflux MSF transporter, Bcr/CflA family (cflA)	Plasmids	pALWED1.8, pALWVS1.4, pALWED1.1	N/A	aadA27 on pALWED1.8. cflA on pALWVS1.4, tet(H) on pALWED1.1	N/A
Zhang 2023	Heihe River basin, northwestern China	S. maltophilia	32	Aminoglycosides (AAC(6’)-Iz and APH(3’)-Iic), aminocoumarins (alaS and mdtC), fluoroquinolones (emrR and mfd), antibacterial free fatty acids (farB), β-lactam (L1 β-lactamase), macrolides (macA and macB), nitroimidazoles (msbA), triclosan (gyrA and TriC), fosfomycin (murA), penams (mecA), peptides (rosB), elfamycins (EF-Tu), pleuromutilins (TaeA), and multidrug resistance (adeA, adeC, adeG, mexJ, mexK, mexW, oprN, oqxA, smeA, smeC, smeD, smeF, smeR, smeS)	N/A	N/A	Transposons	N/A	tnpR. tnpA	N/A	N/A
Gorecki 2021	Siberian active layer	N/A	N/A	N/A	Multidrug resistance	Aminoglycosides (AAC(3)-Ic, AAC(6’)-Isa, AAC(6’)-Ib-SK, AAC(3)-Ib), tetracycline (tetA(48), tetA(60) tet(30), tetS), and sulfonamide(sul4) and two hypothetical beta-lactamases (blaJOHN-1, blaTLA-1)	Plasmids	N/A	N/A	N/A	Moraxellaceae (37.7% of plasmid contigs), Pseudomonadaceae (20.8%), Enterobacteriaceae (20.8%), Pectobacteriaceae (0.9%), and Firmicutes (Family XII; 1.9%), Pseudomonadaceae (37.5%) and Burkholderiaceae (18.8%)
Petrova 2014^23^	N/A	Psychrobacter maritimus MR29-12	N/A	N/A	N/A	N/A	Plasmid	pKLH80	N/A	strA-strB and tetR-tet(H) and blaRTG	Resistance to ampicillin or carbenicillin sensitive to ceftazidime
Haan 2021^12^	Interior Alaska	Bacillus and psedomonas isolates	379	AAC(6)-32, AAC(6’)-Ib7 : aminogycoside inactivating enzyme, AAC(6’)-Ir, bcrC, msbA,emrR, CRP (FosB:encoding an antibiotic inactivating enzyme), (BcII: b-lactamase deactivating penicillins and cephallosporins, soxR, tet(45), BPU-1, TriC, ampC1 beta lactamase, ampH beta lactamase. adeF: efflux pump multidrug resistance, (AbaQ: efflux pump of quinolone drugs), (target alteration: armA, bcrC, (MCR-4.1:colistin), gyrB, PmrF, sgm, and vanJ: conferred resistance to aminoglycosides, peptide, glycopeptide, and fluoroquinolone), KpnE, KpnF, KpnH, BES-1	Antibiotic efflux.	Ampicillin (82.5%), chloramphenicol (51.1%) erythromycin (17.5%) Tetracycline (2.2%).	Plasmids	N/A	N/A	BES-1 beta lactamase, TriC and KpnF antibiotic efflux pumps, and bcrC undecaprenyl pyrophosphate related protein	N/A
Afouda 2020^14^	Unga-Baga-Olonso stream bank										
Nearby the city of Olyokminsk, Siberia	Enterobacter cloacae, Pantoea septica, Pantoea massiliensis, Achromobacter insolitus, Achromobacter spanius, Achromobacter pulmonis, Acinetobacter baumannii, and Janibacter melonis,	394	Most prevalent: AGly, Bla, Flq, rpoB2, EF-Tu, novA, parY and sav1866,	Multidrug Efflux type.	Achromobacter species: acquired resistance to fluoroquinolones. S.epidermidis and S.capitis were resistant to methicillin. The Paenibacillus provencensis isolate: resistant to all tested beta lactams	N/A	N/A	N/A	N/A	N/A	
Costa 2011^15^	Bear Creek, Yukon,	N/A	28	TetM: tetracycline VanX: vancomycin resistance AAC: the aminoglycoside Bla: b-lactamase Erm: f macrolide, lincosamide and type B streptogramin antibiotics	Modify target, protecting it, drug deactivate	B-lactam, tetracycline, aminoglycoside, macrolide lincosamide and type B streptogramin	N/A	N/A	N/A	N/A	N/A
Ren 2022^8^	Thermokarst lakes-Yellow River Source Area	N/A	161	Sediment: rpoB2 (37.7%), rpoB (22.79%), mexQ (3.49%), acrB (2.80%), smeE (2.46%), msbA (2.12%), opmE (2.09%), MexK (1.83%), mexP (1.39%), and MexB (1.34%). Water: rpoB2 (52.76%), rpoB (16.71%), ugd (6.76%), mtrA (3.75%), ileS (3.45%), MexW (2.39%), msbA (1.99%), RanA (1.88%), mdtC (1.24%), and oleC (1.08%)	Target alteration-replacement	Rifamycin (rpoB and rpoB2 genes)-most common Beta-lactam (LMB, TEM, and OXA genes), Tetracycline (tet genes), Mupirocin (ileS and mupB genes), Aminoglycoside (aac genes), vancomycin resistance genes (vanHO, vanRA, and vanYM), aminocoumarin, macrolide, mupirocin, nitroimidazole	N/A	N/A	N/A	N/A	N/A
Perron 2015^19^	N/A	Bacillaceae, Stenotrophomonas, Bacillus	6(active layer) 8(core)	AMK_P_1, AMK_P_2, PEN_P_1 SIS_P_1, SIS_P_2, SIS_P_3, TET_P_1 TET_P_2, AMK_AL_1, AMK_AL_2, DOX_AL_1 DOX_AL_2, PEN_AL_2/CAR_AL_2 SIS_AL_1, SIS_AL_2, TET_AL_1 TET_AL_2	Multidrug-efflux pumps	Aminoglycoside B-lactam tetracycline amikacin	N/A	N/A	N/A	N/A	N/A
Ponder 2005^18^	Kolyma-Indigirka lowland	Arthrobacter sp. 255-12. Exiguobacterium sp. Planococcus, Psychrobacter	N/A	N/A	N/A	Ampicillin, chloramphenicol and tetracycline, streptomycin	N/A	N/A	N/A	N/A	N/A

ARGs, antibiotic resistance genes.

Additionally, our investigation has uncovered numerous diverse pathways that are accountable for the observed antibiotic resistance in permafrost isolates. The mechanisms seen in this study encompassed a range of processes, including efflux pumps and enzymatic modifications of the medicines, most of which offered resistance against multiple classes of antibiotics. The most commonly observed mechanisms are summarized in Table 1.

Furthermore, our study revealed the existence of diverse mobile genetic elements (MGEs), including plasmids and transposons, among the permafrost isolates. The presence of antibiotic-resistance genes was also detected in these mobile elements.

## Discussion

Permafrost, also known as permafrost soil, refers to soil that maintains a temperature of 5–10°C below zero for a duration of at least 2 years. While the majority of permafrost is situated in regions near the North and South Poles, there are instances of permafrost existing at lower latitudes. Permafrost exhibits remarkable stability in its structure, enduring without significant changes for thousands to millions of years. The permafrost environment is deemed extreme due to its subzero temperatures and the presence of background radiation over geological timescales^24,25^.

There is ample evidence available pointing towards the existence of a wide variety of bacteria in permafrost sediments^26,27^. Typically, a shallow active layer of soil above the permafrost thaws during the summer months, which promotes microbial growth and releases previously confined pathogens. Such pathogens result in epidemics that burden already weakened health infrastructures in circumpolar regions^7^. Furthermore, these permafrost sources harbor several ARGs and bacterial virulence factor genes (VFGs), which, if disseminated via horizontal transmission, can pose a serious challenge to pharmacological systems worldwide^28^. Our review has summarized key findings regarding such factors found in permafrost soil.

### Bacteria identified

A considerable quantity of living microorganisms has been extracted from permafrost in various Arctic and Antarctic locations. These encompass aerobic heterotrophs, as well as bacteria capable of nitrogen fixation, sulfur oxidation, sulfur reduction, and anaerobic processes. These microorganisms exhibit psychrotrophic, mesophilic, and thermophilic characteristics^29^. Some of the strains isolated from permafrost sediments include *Staphylococcus warneri* and *Staphylococcus hominis* ^13^, *Psychrobacter psychrophilus*, and *Psychrobacter maritimus* ^18,20,23^, *Pseudomonas aeruginosa* ^12,21^, *Acinetobacter spp* ^14,16^, *Achromobacter spp* ^14^, *Stenotrophomonas maltophilia* ^17^, and *Bacillus spp* ^18,19^. Such strains did not differ significantly from their modern clinical and environmental counterparts in terms of genetic content. There are several strategies that these bacterial genera may employ for survival in harsh permafrost conditions, such as increased reliance on scavenging detrital biomass, chemotaxis, dormancy by forming spores or biofilms, environmental sensing, and stress response^30^.

### Antibiotic resistance genes (ARGs) and mechanisms identified, and their historical development

Bacteria have evolved sophisticated mechanisms of drug resistance to avoid killing by antimicrobial molecules. A bacterium can typically achieve resistance to a specific antimicrobial class through various biochemical pathways. A single bacterial cell may employ a variety of resistance mechanisms to survive the impact of an antibiotic. The emergence of these resistance mechanisms is attributed to the remarkable genetic adaptability of bacteria, enabling them to respond to a diverse range of environmental threats, including antibiotics that may pose a risk to their survival. This adaptability occurs through two main processes: 1) mutations in genes often associated with the compound’s mechanism of action, and 2) the incorporation of foreign DNA containing resistance determinants through horizontal gene transfer (HGT)^31^.

An important concept regarding antibiotic resistance is that it is not a recent phenomenon but one that has ancient roots. Munita *et al*. stated that most antimicrobial compounds are naturally produced molecules, and, as such, co-resident bacteria have evolved mechanisms to overcome their action in order to survive. Thus, these organisms are often considered to be ‘intrinsically’ resistant to one or more antimicrobials; it is these intrinsically resistant bacteria which then result in the spread of antibiotic resistance via horizontal transmission^31^. The presence of ARGs and their phenotypes in ancient bacteria liberated from permafrost thawing is strong evidence of the aforementioned concept that antibiotic resistance dates back to ancient times since 1) these permafrost sediments have persisted for thousands of years, 2) bacteria trapped inside them are found to be preserved for long periods of time in their dormant forms, and 3) genomic sequencing reveals a considerable similarity between ancient and modern counterparts of such strains.

Several studies were conducted which used targeted metagenomic sequencing on ancient bacteria discovered in permafrost sediments dating as back as 3.5 million years old. These studies identified a wide variety of ARGs encoding resistance via mechanisms such as multidrug efflux pumps and modification of antimicrobial molecules, to a wide range of antibiotics, mainly tetracyclines, beta-lactams, fluroquinolones, macrolides, aminoglycosides, and glycopeptides. Interestingly these antibiotics did not exist at the same time as the creation of these permafrost sediments. Moreover, these genes and their corresponding mechanisms are also found in modern-day strains of the same species. This evidence suggests that antibiotic resistance is a natural phenomenon that predates the modern selective pressure of clinical antibiotic use^8,13–15,19^. Of these studies, Ren and Luo^8^ found rifamycin resistance genes to be the most abundant while also discovering that both water and soil sediments, formed by permafrost thawing, harbor ARGs, with a higher proportion present in the latter. Costa *et al*.^15^ identified a complete vancomycin resistance element *vanA*, which is similar to the modern variants.

Rakitin *et al*.^16^ performed a genome analysis of five strains of *Acinetobacter lwoffii* isolated from permafrost aged from 15 thousand to 1.8 million years and compared the ARGs present in their plasmids to the ones found in modern counterparts. While there were no significant differences in their genetic content, the permafrost strains harbored lower levels of ARGs than modern clinical isolates. The authors suggest that this difference may be due to the fact that the permafrost strains have not been exposed to the same selective pressures as modern clinical isolates. A similar study conducted by Zhang *et al*.^17^ discusses the antibiotic and metal resistance of *Stenotrophomonas maltophilia* isolates from the Eboling permafrost of the Tibetan Plateau. The authors found that the permafrost *Stenotrophomonas maltophilia* strains were resistant to a variety of antibiotics and metals, including chloramphenicol, trimethoprim-sulfamethoxazole, erythromycin, Zn2+, Ni2+, Cu2+, and Cr6+. However, the permafrost strains had lower minimum inhibitory concentrations (MICs) and less diversity of antibiotic and metal resistance genes (ARGs and MRGs) than clinical *Stenotrophomonas maltophilia* strains. This difference may be attributed to the absence or presence of only one kind of ARG or MRG on a single genomic island due to the lack of selective pressures commonly found in modern human-impacted environment. The results also indicated that the co-occurrence of antibiotic and metal resistance was due to a certain innate ability of *Stenotrophomonas maltophilia*. Both these studies suggest that while antibiotic resistance mechanisms are of ancient origin, they are continuously evolving and expanding, becoming increasingly diverse and abundant.

Haan *et al*.^12^ explored an additional outcome as to how soil disturbance and subsequent changes in community composition impact ARGs in the active layer resistome. The findings reveal a high proportion of isolates resistant to tested antibiotics, particularly ampicillin. ARG abundance and the proportion of resistant isolates increased with disturbance, but ARGs per isolate were more influenced by phylogeny than isolation site. Compared to a global soil bacteria database (RefSoil+), the authors’ isolates within the same genera exhibited distinct ARGs, with a higher prevalence on plasmids. These results underscore that both phylogeny and ecology contribute to shaping the resistome, indicating that a disturbance-induced shift in community composition will likely be reflected in the prevalent ARGs in the active layer resistome.

### Mobile genetic elements (MGEs) identified

MGEs broadly refer to any genetic element capable of moving itself, with or without duplication, from one site in a genome to another, or from one cell to another. In prokaryotes, these include insertion sequences, transposons, gene cassettes/integrons, and those that are able to transfer between bacterial cells, such as plasmids and integrative conjugative elements. Together, these elements play a central role in facilitating horizontal genetic exchange and, therefore, promote the acquisition and spread of resistance genes^32^. The bacteria discovered in permafrost isolates also house several MGEs containing various ARGs, which corroborates with the claim that antibiotic resistance is an ancient phenomenon.

A study conducted by Petrova *et al*.^20^ identified multidrug-resistant plasmid, pKLH80, and transposon Tn5080, isolated from the ancient permafrost bacterium *Psychrobacter psychrophilus*, which contain genes encoding resistance to streptomycin and tetracycline, and mobile elements including an insertion sequence (ISPpy1) belonging to IS3 family. Another study^23^ revealed similar findings and described the pKLH80 plasmid as having several functional regions, including a replication module, a mobilization module, and an accessory region containing antibiotic resistance genes and insertion sequences. Petrova *et al*.^21^ also reported the discovery of a Tn5045 transposon isolated from an ancient permafrost *Pseudomonas spp.* This is a composite transposon containing three distinct elements: a Tn1013-like Tn3 family transposon, a class 1 integron, and a TnOtChr-like Tn3 family transposon. The class 1 integron, as claimed by the author, is the first finding of an ancient integron containing antibiotic-resistance genes. It is termed InC*, is inserted into the Tn1013* res-region, and contains a) 50-CS located integrase gene, b) 30-CS located qacEΔ1 and sulfonamide resistance sulI genes, c) a single cassette encoding the streptomycin resistance, aadA2-gene. Rakitin *et al*.^16^ also discovered several ARGs on different plasmids isolated from five permafrost strains of *Acinetobacter lwoffii*; an aadA27 ARG on plasmid pALWED1.8, cflA on pALWVS1.4, and tet(H) on pALWED1.1. Interestingly, similar MGEs were discovered in their modern clinical counterparts as well.

### Effect on global healthcare

Permafrost thawing releases several viable strains of microbes against which present living organisms, including humans, have not yet developed immunity. Such strains may result in epidemics in nearby circumpolar regions. A well-known example is that of *Bacillus anthracis* since its spores can endure harsh freezing conditions; in 2016, there was an anthrax outbreak in Siberia attributed to contact with frozen carcasses in permafrost, as the permafrost melted, the anthrax spores were released which led to the unfortunate outcome of one person and more than 2000 reindeer succumbing to illness after ingesting the spores^33^. Another study conducted by Revich *et al*. discussed the potential role of climate change in causing such zoonotic infection epidemics; gradual temperature increases result in permafrost melting, which exposes zoonotic bacteria such as *Leptospira interrogans*, *Brucella spp.*, and *Francisella tularensis* to various arctic animals, including vectors for these bacteria, which migrate to nearby regions disseminating the pathogen^34^. Additionally, the prevalence of ARGs in these permafrost isolates, as discussed previously, may aggravate such bacterial disease outbreaks since modern antibiotics would face difficulty in eradicating them^8,13–15,19^.

This phenomenon may also cause viral epidemics. A study conducted by Hofmeister *et al*. discusses how permafrost thawing, and subsequent release of viruses may have played a role in influenzavirus and coronavirus outbreaks in Northern Hemispheric regions^6^. The study also discusses that these viruses are then linked with human exposure via long-distance atmospheric transport, which may be facilitated by air pollutant particulates^6^. Influenzavirus, in particular, is disseminated via various biotic and abiotic means, a common biotic example being aquatic birds^7^. Permafrost may also be the source of calicivirus and enteroviruses (e.g. poliovirus, echovirus, and coxsackievirus) outbreaks in circumpolar regions since their permafrost deposits have been discovered and they are readily transmitted by contaminating water sources^7^.

In addition to bacteria and viruses, permafrost may also harbor several fungal species, such as *Geomyces spp.*, *Cladosporium spp.*, *Aspergillus spp.*, and *Penicillium spp* ^35^, which have been found to cause diseases in certain plant and insect species in Siberia^36^. Apart from causing infectious diseases, permafrost melting may also burden the global healthcare system by causing several respiratory disorders. When frozen soil thaws, cold-adapted microbes encounter increased soil water content from melted ice and enriched nutrients from growing vegetation. This prompts microbial growth and fermentation, releasing greenhouse gases into the atmosphere which pose potential risks to human health, such as asthma and respiratory failure^4^.

### Limitations

However, this present narrative review is not without its limitations. For instance, the selected search timeframe, ending in December 2023, may exclude newer research. Moreover, imposing language limitations potentially omits relevant studies published in non-English languages. Furthermore, including all publication types without quality assessment could incorporate less rigorous studies. Finally, the focus solely on viral and bacterial pathogens might overlook potential risks from other types of microbes released by permafrost thaw.

## Conclusion

Climate change-driven permafrost thawing presents a major health concern, it releases pathogens and contributes to worsening antibiotic resistance. Our literature review, adhering to PRISMA guidelines, included 13 studies on antibiotic resistance genes in permafrost isolates. Our results revealed diverse bacterial genera, resistance mechanisms, and MGEs housing resistance genes. Given the gravity of this emerging global health challenge, concerted efforts are crucial for effective mitigation.

## Ethical approval

Ethics approval was not required for this review.

## Consent

Informed consent was not required for this review.

## Source of funding

None.

## Author contribution

M.M.S.: project administration, supervision, validation, and writing – original draft; N.E. and R.A.: data curation, investigation, and writing – original draft; A.G.: visualization, resources, and writing – original draft; M.A.: supervision, validation, and writing – reviewing and editing; A.E.: conceptualization, project administration, and methodology; H.K.: resources, software, and writing – reviewing and editing; M.H.: funding acquisition (100% waiver), visualization, and resources.

## Conflicts of interest disclosure

The authors declare that they have no financial conflict of interest with regard to the content of this report.

## Research registration unique identifying number (UIN)

1. Name of the registry: not applicable.
2. Unique identifying number or registration ID: not applicable.
3. Hyperlink to your specific registration (must be publicly accessible and will be checked): not applicable.

## Guarantor

Muhammad M. Saleem.

## Data availability statement

No new data was generated or analyzed in this paper.

## Provenance and peer review

Not invited.
